# Kinetoplastid kinetochore proteins KKT14–KKT15 are divergent Bub1/BubR1–Bub3 proteins

**DOI:** 10.1098/rsob.240025

**Published:** 2024-06-12

**Authors:** Daniel Ballmer, William Carter, Jolien J. E. van Hooff, Eelco C. Tromer, Midori Ishii, Patryk Ludzia, Bungo Akiyoshi

**Affiliations:** ^1^ Department of Biochemistry, University of Oxford, Oxford OX1 3QU, UK; ^2^ The Wellcome Centre for Cell Biology, Institute of Cell Biology, University of Edinburgh, Edinburgh EH9 3BF, UK; ^3^ Laboratory of Microbiology, Department of Agrotechnology and Food Sciences, Wageningen University and Research, 6708 HB Wageningen, The Netherlands; ^4^ Cell Biochemistry, Groningen Biomolecular Sciences and Biotechnology Institute, Faculty of Science and Engineering, University of Groningen, 9747 AG Groningen, The Netherlands

**Keywords:** kinetochore, chromosome segregation, spindle checkpoint, kinetoplastid, *Trypanosoma brucei*

## Abstract

Faithful transmission of genetic material is crucial for the survival of all organisms. In many eukaryotes, a feedback control mechanism called the spindle checkpoint ensures chromosome segregation fidelity by delaying cell cycle progression until all chromosomes achieve proper attachment to the mitotic spindle. Kinetochores are the macromolecular complexes that act as the interface between chromosomes and spindle microtubules. While most eukaryotes have canonical kinetochore proteins that are widely conserved, kinetoplastids such as *Trypanosoma brucei* have a seemingly unique set of kinetochore proteins including KKT1–25. It remains poorly understood how kinetoplastids regulate cell cycle progression or ensure chromosome segregation fidelity. Here, we report a crystal structure of the C-terminal domain of KKT14 from *Apiculatamorpha spiralis* and uncover that it is a pseudokinase. Its structure is most similar to the kinase domain of a spindle checkpoint protein Bub1. In addition, KKT14 has a putative ABBA motif that is present in Bub1 and its paralogue BubR1. We also find that the N-terminal part of KKT14 interacts with KKT15, whose WD40 repeat beta-propeller is phylogenetically closely related to a direct interactor of Bub1/BubR1 called Bub3. Our findings indicate that KKT14–KKT15 are divergent orthologues of Bub1/BubR1–Bub3, which promote accurate chromosome segregation in trypanosomes.

## 1. Introduction

Accurate transmission of genetic material from mother to daughter cells is essential for the survival of all organisms. Segregation of replicated chromosomes in eukaryotes is achieved by a macromolecular protein complex called the kinetochore, which links centromeric DNA to microtubules [1,2]. Once all chromosomes have achieved proper attachments, the multi-subunit E3 ubiquitin ligase complex called the anaphase-promoting complex/cyclosome (APC/C) gets activated. This leads to the degradation of anaphase inhibitors securin and cyclin B, triggering sister chromatid separation and exit from mitosis [3,4].

The spindle checkpoint is a surveillance system that monitors defects in kinetochore–microtubule attachments and delays the onset of anaphase [5]. It works by inhibiting the activity of the APC/C that is in complex with its co-activator protein Cdc20. Spindle checkpoint components include Mps1, Bub1, BubR1 (Mad3), Bub3, Mad1 and Mad2 [6–8]. The mitotic checkpoint complex is a potent inhibitor of APC/C^Cdc20^, which in humans consists of Mad2, Cdc20, BubR1 and Bub3 [9]. Unattached kinetochores recruit these checkpoint proteins to catalyse the formation of the mitotic checkpoint complex [10]. Kinetochore recruitment of spindle checkpoint proteins therefore needs to be under tight control. Bub3, a WD40 repeat domain protein, directs Bub1 and BubR1 (Mad3) to kinetochores by recognizing the Mps1-phosphorylated MELT motif of the outer kinetochore protein KNL1 [11–15]. Bub1 and BubR1 are paralogous proteins [16], which have a kinase and pseudokinase domain in their C-terminus, respectively, while Mad3, a Bub1 paralog in yeast, does not have a kinase domain. The kinase activity of Bub1 is largely dispensable for its spindle checkpoint function [17,18]. The pseudokinase domain in human BubR1 is thought to play a role in ensuring its protein stability [16]. Bub1, BubR1 and Mad3 carry the Gle2-binding sequence (GLEBS) motif that binds Bub3 and the ABBA motif that interacts with Cdc20 [19–22].

Despite its importance in ensuring accurate chromosome segregation, some organisms (e.g. yeasts, fruit flies and human HAP1 cells) do not require the spindle checkpoint for their proliferation or development under normal conditions [23–27]. It is thought that these organisms do not require a feedback-induced mitotic delay because all chromosomes can establish proper kinetochore–microtubule attachments before the APC/C gets fully activated. Furthermore, spindle checkpoint components are apparently absent in some organisms, including *Trypanosoma brucei*, which cannot halt cell cycle progression in response to spindle defects [28–32]. *Trypanosoma brucei* is an experimentally tractable parasite that belongs to kinetoplastids, a class of unicellular flagellated eukaryotes that are highly divergent from traditional model eukaryotes [33–35]. They include the parasitic order Trypanosomatida (e.g. *T. brucei*, *Trypanosoma cruzi* and *Leishmania*), free-living Bodonida (e.g. *Bodo saltans*), both belonging to the subclass Metakinetoplastina, and the subclass Prokinetoplastina (e.g. *Apiculatamorpha spiralis*, *Papus ankaliazontas* and *Perkinsela*) [36]. Very little is known about how kinetoplastids regulate cell cycle progression or ensure accurate chromosome segregation without a functional spindle checkpoint.

Like spindle checkpoint components, kinetochore proteins are widely conserved among eukaryotes [37,38]. However, unique kinetochore proteins called Kinetoplastid KineTochore 1–25 (KKT1–25) and KKT-Interacting Protein 1–12 (KKIP1–12) are present in *T. brucei* [39–43]. These proteins are conserved among kinetoplastids but do not have a significant sequence similarity to canonical kinetochore proteins, meaning that they are attractive drug targets against diseases caused by kinetoplastid parasites [44,45]. Understanding the structure and function of kinetoplastid kinetochore proteins also has the potential to shed light on fundamental requirements for the chromosome segregation machinery in eukaryotes.

In this study, we focus on KKT14 and KKT15, proteins of unknown functions that localize at the kinetochore from G2 until the end of anaphase in *T. brucei* [39]. Previous bioinformatics analysis using advanced hidden Markov model (HMM) searches (e.g. HMMER and HHpred) of *T. brucei* KKT14 failed to identify any obvious conserved domain. KKT15 has WD40 repeats that likely form a beta-propeller, a domain found in many different proteins, including Bub3, Cdc20 and mRNA export factor Rae1/Gle2 [46]. Here, we discover that KKT14 has a pseudokinase domain in its C-terminus, which is most similar to the kinase domain of Bub1. We also identify a putative ABBA motif in KKT14. The N-terminal part of KKT14 interacts with KKT15, which we suggest to be a Bub3 orthologue. These results reveal that kinetoplastids possess divergent Bub1/BubR1 and Bub3 proteins.

## 2. Results

### 2.1. Crystal structure of KKT14 C-terminal domain reveals similarity to Bub1

**Figure 1.**
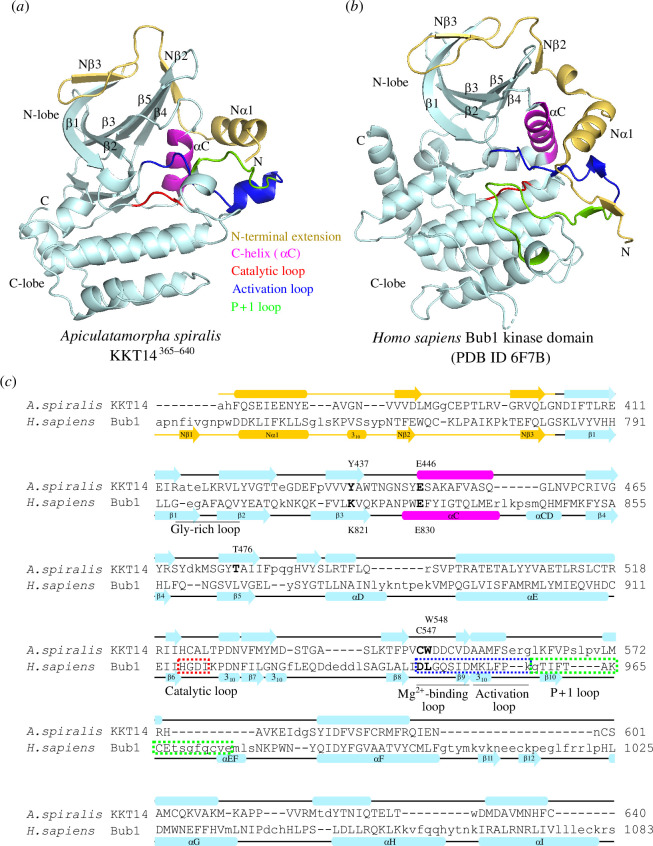
Crystal structure of *A. spiralis* KKT14 reveals similarity to the Bub1 kinase domain. (*a*,*b*) Cartoon representation of *A. spiralis* KKT14^365–640^ (*a*) and human Bub1 kinase domain (PDB accession 6F7B [47]) (*b*). The fold nomenclature of the N-terminal extension and the kinase domain of Bub1 is based on [48]. (*c*) Structure-based pairwise alignment of *A. spiralis* KKT14 and human Bub1 kinase domain based on the DALI search output. Structurally equivalent residues are in uppercase, while structurally non-equivalent residues (e.g. in loops) are in lowercase. Secondary structures were assigned using DSSP [49].

To gain insights into the function and evolutionary origin of KKT14, we aimed to obtain its high-resolution structural information. By screening four kinetoplastid species (*T. brucei*, *T. cruzi*, *Paratrypanosoma confusum* and *Apiculatamorpha spiralis*), we succeeded in determining a 2.2 Å resolution crystal structure for the C-terminal domain of the KKT14 protein from the prokinetoplastid *A. spiralis* (clone PhF-6) (figure 1*a*, table 1 and electronic supplementary material, table S1). *Apiculatamorpha spiralis* KKT14^365−640^ crystallized with two molecules in an asymmetric unit. Both molecules were essentially identical except for minor variations in flexible loops.

**Table 1.** Data collection and refinement statistics for *Apiculatamorpha spiralis* KKT14^365−640^.

data collection	
beamline	Diamond Light Source I03
wavelength (Å)	0.9763
space group (Z)	*P 1 2*_1_ *1*
unit cell	49.76 Å 87.58 Å 71.88 Å 90^◦^ 93.61^◦^ 90^◦^
resolution range (Å)	49.66–1.95 (1.95–1.99)
unique reflections	41 803 (2243)
completeness (%)	93.3 (100.0)
multiplicity	6.7 (6.7)
I/I*σ*	6.9 (0.7)
R*meas*	0.188 (2.640)
CC1*/*2	1.0 (0.6)
Wilson B-factor (Å^2^)	30.14
**refinement**	
no. reflections	28 797 (1146)
R*work*	0.22 (0.36)
R*free*	0.24 (0.40)
number of atoms	4571
protein	4308
solvent	263
RMS bonds (Å)	0.009
RMS angles (^◦^)	1.23
Ramachandran favoured (%)	96.13
Ramachandran allowed (%)	3.87
Ramachandran outliers (%)	0.00
average B-factor (Å^2^)	39.00

*Notes:* Values in parentheses correspond to the highest resolution shell.

Interestingly, a structural homology search using the distance-matrix alignment (DALI) server [50] revealed similarity to a protein kinase fold with an N-lobe and C-lobe (figure 1*a* and electronic supplementary material, table S2). The N-lobe contains a five-stranded β-sheet, a helix termed the C-helix (αC), and loops that correspond to the catalytic loop and activation loop, while the C-lobe comprises a bundle of α-helices (figure 1*a*). Although most protein kinases share a similar fold [51], we found that the most similar structure of *A. spiralis* KKT14^365−640^ in the PDB database was the Bub1 kinase domain (figure 1*b*). Human Bub1 was the top hit in the DALI search with a Z-score of 15.5, while the next best hit was the MST3 kinase (Z-score 13.0) (electronic supplementary material, table S2). We obtained a similar result for an AlphaFold2-predicted structure of *T. brucei* KKT14^358–685^ (electronic supplementary material, figure S1*a*), showing a Z-score of 15.9 for human Bub1 and 12.9 for the next best hit, the PAK3 kinase (electronic supplementary material, table S2). Moreover, searches with Foldseek against the AlphaFold2-predicted structure database [52] produced congruent results (see §4). The higher structural similarity of KKT14 to the kinase domain of Bub1 rather than other kinases is owing to an N-terminal extension that is present in Bub1 and KKT14 (figure 1*a*–*c*; electronic supplementary material, table S2) [47,48,53–55]. These results show that the C-terminal domain of KKT14 has a kinase fold with the most similar structure being the Bub1 kinase domain, raising a possibility that KKT14 is a Bub1/BubR1 orthologue.

**Figure 2.**
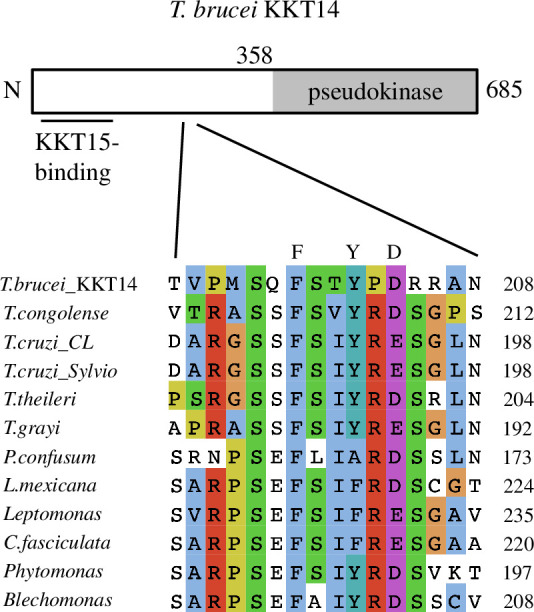
KKT14 has a putative ABBA motif. Schematic of the *T. brucei* KKT14 protein and a multiple sequence alignment showing a putative ABBA motif (consensus Fx[ILV][FHY]x[DE] based on [19]) conserved in trypanosomatid KKT14 proteins.

Besides a kinase/pseudokinase domain, Bub1 and BubR1 have various conserved domains and motifs [19,20,22,56]. We identify a putative ABBA motif (consensus: Fx[ILV][FHY]x[DE]) in KKT14, which is highly conserved among trypanosomatids (figure 2), as well as KEN boxes in some kinetoplastids (electronic supplementary material, figure S2). In contrast, other domains such as a TPR, CDI or a KARD domain were not found. The presence of an ABBA motif and a C-terminal Bub1-like kinase fold strongly supports the possibility that KKT14 is a divergent Bub1-like protein.

### 2.2. KKT14 C-terminal domain is an inactive pseudokinase

**Figure 3.**
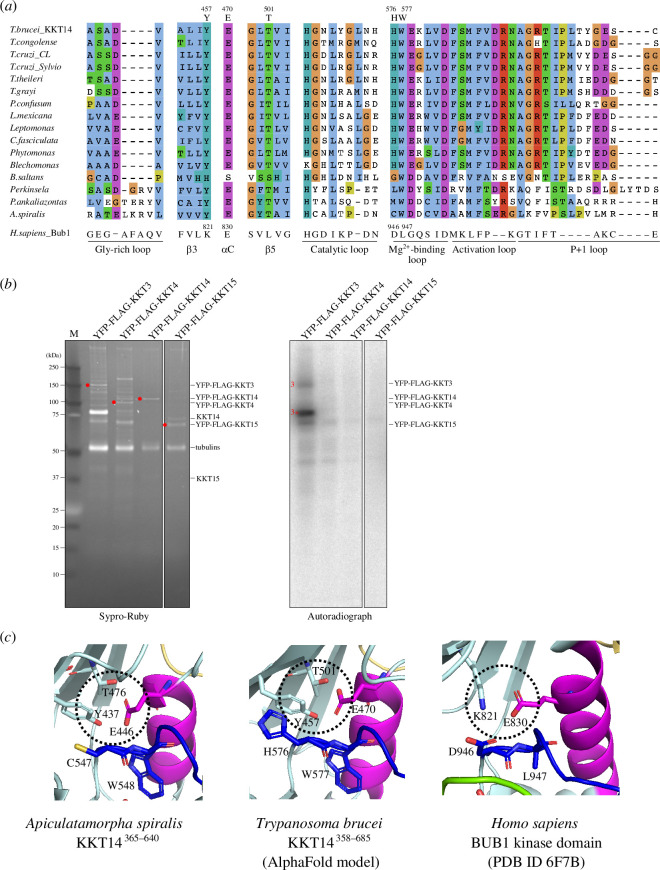
KKT14 lacks key residues that are present in active protein kinases. (*a*) Multiple sequence alignment of kinetoplastid KKT14 sequences highlighting the regions that correspond to the key parts of the Bub1 kinase domain. Note that T501 and W577 in *T. brucei* are conserved among kinetoplastids. (*b*) Lack of detectable auto-phosphorylation activity for KKT14. Indicated proteins were immunoprecipitated from trypanosomes using FLAG antibodies and eluted with FLAG peptides. The left panel shows a Sypro-Ruby stained SDS-PAGE gel (red circles indicate FLAG-tagged proteins), while the right panel shows phosphorylation detected by autoradiography. A degradation product of YFP-FLAG-KKT3 is indicated by 3*. (*c*) The C-helix has an ‘in’ conformation in the *A. spiralis* KKT14 crystal structure and AlphaFold2-predicted *T. brucei* KKT14 structure (electronic supplementary material, dataset S1). Note that E446 in the C-helix is in close proximity with Y437 of β3 and T476 of β5 in *A. spiralis* (E470, T501 and Y457 in *T. brucei,* respectively), while E830 in the C-helix forms a salt bridge with the conserved β3 lysine (K821) in Bub1.

Despite the structural similarity, however, a structure-based sequence alignment of KKT14 with Bub1 shows a lack of conserved residues that play key roles in catalytically active protein kinases (figure 1*c*). Most notably, none of KKT14 orthologues in kinetoplastids has a conserved β3 lysine (K821 in Bub1) (figure 3*a*), whose mutation results in an inactive kinase [57,58]. In addition, KKT14 does not appear to have the Gly-rich motif (GxGxxG in most kinases, which is essential for stabilizing ATP phosphates during catalysis), and the HRD motif in the catalytic loop (usually His-Arg-Asp: HGD in Bub1) is HGN in *T. brucei* and HCA in *A. spiralis* (figure 3*a*). Furthermore, the DFG motif (usually Asp-Phe-Gly: DLG in Bub1), which is required for Mg^2+^ coordination, is HWE in *T. brucei* and CWD in *A. spiralis* KKT14 (figure 3*a*). Given that even a single amino acid change of key residues in these motifs results in inactive kinases [59], these findings strongly suggest that the C-terminal domain of KKT14 is a catalytically inactive pseudokinase. Consistent with this possibility, KKT14 purified from trypanosomes did not have any detectable auto-phosphorylation activity, while the KKT3 kinase did (figure 3*b*).

Although KKT14 appears to be an inactive pseudokinase, features of active protein kinase conformations are found in its structure. In the active conformation of protein kinases, the DFG motif and the C-helix have an ‘in’ conformation, where the conserved phenylalanine or leucine of the DFG motif (L947 in Bub1) points out of the active site and the aspartic acid (D946 in Bub1) faces the ATP-binding site (figure 3*c*) [57]. In addition, the β3 lysine (K821 in Bub1) forms a salt bridge with the glutamate (E830 in Bub1) in the C-helix (figure 3*c*). In the crystal structure of *A. spiralis* KKT14, W548 (the phenylalanine equivalent of the DFG motif, which is CWD in *A. spiralis* KKT14), strictly conserved among kinetoplastids, faces away from the active site. Even though KKT14 in almost all kinetoplastids has the conserved glutamate in the C-helix (E446 in *A. spiralis*), the position of β3 lysine has tyrosine (Y437 in *A. spiralis*). Nonetheless, the C-helix sits close to β3, mediated by apparent interactions among Y437, E446 and T476 in β5. Similar interactions were observed in the AlphaFold2-predicted structure of *T. brucei* KKT14 (figure 3*c*), and the threonine is strictly conserved among kinetoplastids (T501 in *T. brucei*) (figure 3*a*). We also note that the positions of the N-terminal extension, C-helix, catalytic loop and Mg^2+^-binding loop are very similar in between the crystal structure of *A. spiralis* KKT14 and the AlphaFold2-predicted structure of *T. brucei* KKT14 (electronic supplementary material, figure S1), despite a limited similarity between the KKT14 sequences of the two species (20.1% identical, 31.5% similar). These findings suggest that the catalytically inactive pseudokinase domain of KKT14 takes an active-like conformation of a kinase fold.

### 2.3. KKT15 is a divergent Bub3 protein

**Figure 4.**
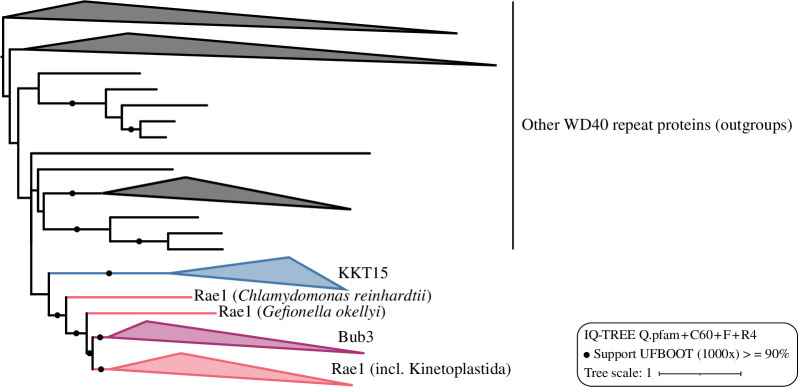
Phylogeny of KKT15 and related WD40 repeat proteins. Subtree of the full phylogeny presented in electronic supplementary material, figure S3*a*. Note that in the alignment approach applied here, KKT15 proteins were prompted to form a single group (see §4).

Our previous sequence similarity searches were unsuccessful in finding homology between *T. brucei* KKT14 and Bub1/BubR1. Using HHpred [60] with a sensitive alignment of KKT14 proteins from an extended kinetoplastid dataset (electronic supplementary material, table S3), we were now able to observe a link to human Bub1, albeit with a non-significant *E*-value (electronic supplementary material, table S4). Owing to the low level of sequence similarities, conducting a phylogenetic analysis was not feasible. In contrast, searches with a KKT15 alignment retrieved many WD40 domain proteins with highly significant *E*-values and had Bub3 as its best hit (electronic supplementary material, table S4). The WD40 beta-propeller is a domain present in Bub3 and many other proteins [46]. To investigate to which WD40 group KKT15 might belong, we conducted a phylogenetic analysis using two multiple sequence alignments of WD40 proteins (see §4). In both cases, KKT15 orthologues cluster with Bub3 and Rae1, which are known to be closely related to one another [38]. One alignment yields all KKT15 orthologues as sisters to Bub3 and Rae1 (figure 4 and electronic supplementary material, figure S3*a*), whereas the other places only a particular subset, those of *B. saltans*, *A. spiralis* and *P. ankaliazontas*, next to Bub3 and Rae1 (electronic supplementary material, figure S3*b*), suggesting that trypanosomatid KKT15 sequences differ more from Bub3/Rae1 than those of bodonids and prokinetoplastids. Importantly, KKT15 proteins do not cluster within Bub3 in either tree, so we cannot unequivocally designate them as Bub3 orthologues. However, the fact that kinetoplastids have clear Rae1 orthologues (electronic supplementary material, figure S3*a,b*) [61], in combination with KKT15’s kinetochore localization and interaction with KKT14 (see below), analogous to Bub1/BubR1-Bub3, prompts us to propose that KKT15 is a Bub3 orthologue.

### 2.4. KKT14 interacts with KKT15

**Figure 5.**
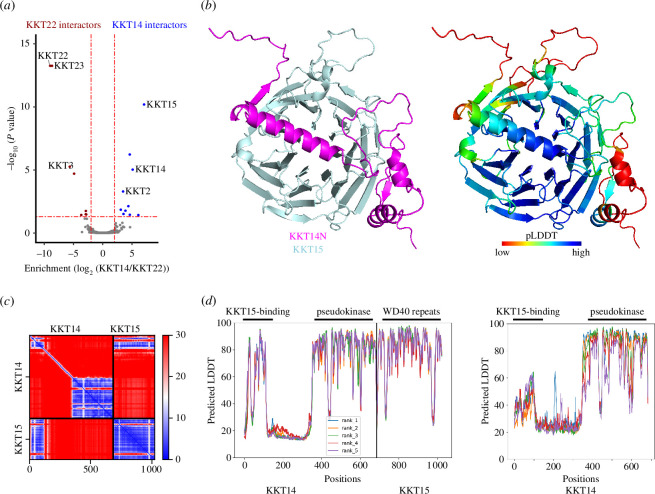
KKT14 is predicted to interact with KKT15 directly. (*a*) A volcano plot showing relative enrichment and significance values between the immunoprecipitates of YFP-KKT14 and YFP-KKT22 (*n* = 4 each). KKT22 was used as a comparison, which mainly co-purifies with KKT23 and KKT3 [43]. See electronic supplementary material, table S5, for all proteins identified by mass spectrometry. (*b*) AlphaFold2 predictions of the KKT14^2–125^–KKT15 complex in cartoon representation (electronic supplementary material, dataset S2). (*c*) The PAE plots for the rank 1 model for KKT14–KKT15, predicting interactions via the N-terminal part of KKT14. (*d*) pLDDT plots for KKT14–KKT15 (left) and KKT14 (right). AlphaFold2-predicted models are provided in the electronic supplementary material, datasets S3 and S4. PAE, predicted aligned error.

KKT14 and KKT15 localize at kinetochores from G2 to anaphase [39]. This localization pattern differs from the rest of transiently localized kinetoplastid kinetochore proteins that start to localize at kinetochores from the S phase, suggesting that KKT14 and KKT15 may directly interact with each other. This possibility is supported by our mass spectrometry analysis (figure 5*a*). Although KKT14 does not appear to have a GLEBS motif present in the N-terminal region of Bub1/BubR1 that interacts with Bub3 [12,21,62], AlphaFold2 predicted an interaction between the N-terminal region of KKT14 and KKT15 (figure 5*b*,*c*). The region of KKT14 predicted to interact with KKT15 is well conserved among trypanosomatids (residues 2–111 in *T. brucei*) (electronic supplementary material, figure S2). Interestingly, the confidence score (predicted local distance difference test: pLDDT) for this region improved when predicted as a KKT14–KKT15 complex, compared with KKT14 alone (figure 5*d*), implying that this region of KKT14 is more likely to form a secondary structure (α-helices) in the complex. These results strongly support the idea that kinetoplastid kinetochore proteins KKT14 and KKT15 are divergent Bub1/BubR1 and Bub3 proteins, although they might have adopted a distinct interaction mode.

**Figure 6.**
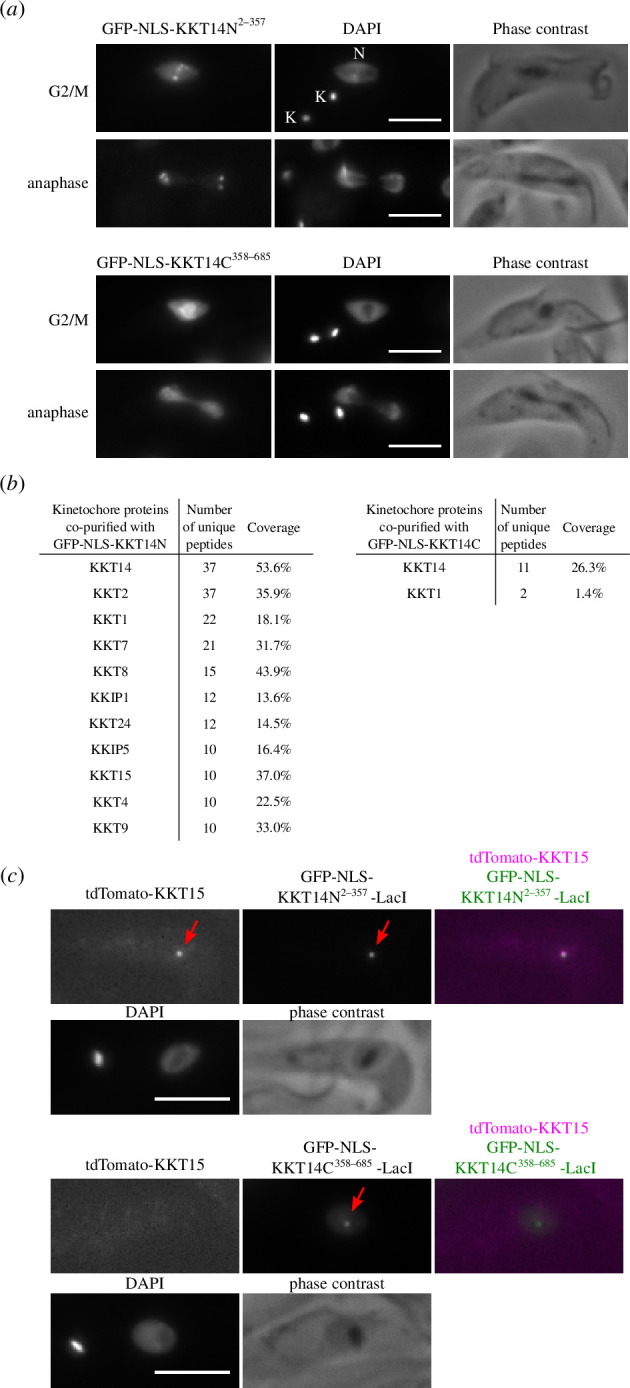
N-terminal region of KKT14 binds KKT15. (*a*) Ectopically expressed GFP-KKT14N^2–357^ localizes at kinetochores, while GFP-KKT14C^358–685^ does not. K and N stand for the kinetoplast (mitochondrial DNA) and nucleus, respectively. Cell cycle stages of individual cells were determined based on the number of K and N as described previously [63,64]. The GFP fusion proteins were expressed in trypanosomes using 10 ng ml^−1^ doxycycline for 1 day and fixed for microscopy. Cell lines: BAP2386, BAP2387. (*b*) Immunoprecipitation/mass spectrometry analysis shows that GFP-KKT14N co-purifies with many kinetochore proteins, while GFP-KKT14C does not. Immunoprecipitation was carried out using cells expressing the GFP fusion proteins using 10 ng ml^−1^ doxycycline for 1 day. See electronic supplementary material, table S5, for all proteins identified by mass spectrometry. (*c*) KKT14N^2–357^ is sufficient to recruit KKT15 in trypanosomes. Recruitment of tdTomato-KKT15 was observed in 100% or 0% of 1K1N (G1) cells that have GFP-KKT14N^2–357^-LacI or GFP-KKT14C^358–685^-LacI dots, respectively (*n* = 10 each). The GFP fusion proteins were expressed in trypanosomes using 10 ng ml^−1^ doxycycline for 1 day. Cell lines: BAP2655, BAP2656. Scale bars, 5 µm.

To better characterize KKT14, we ectopically expressed its fragments in trypanosomes. We found that KKT14N^2–357^ localized at kinetochores from G2 to anaphase, while KKT14C^358–685^ only had diffuse nuclear signals (figure 6*a*). Immunoprecipitation of these fragments revealed that KKT14N co-purified with many kinetochore proteins, including KKT15 (figure 6*b* and electronic supplementary material, table S5). Furthermore, LacO/LacI-based tethering experiments show that KKT14N, not KKT14C, was able to recruit KKT15 to an ectopic locus *in vivo* (figure 6*c*). These results suggest that the N-terminal region of KKT14 interacts with KKT15, as predicted by AlphaFold2 (figure 5*b*).

### 2.5. KKT14 and KKT15 are required for accurate chromosome segregation

**Figure 7.**
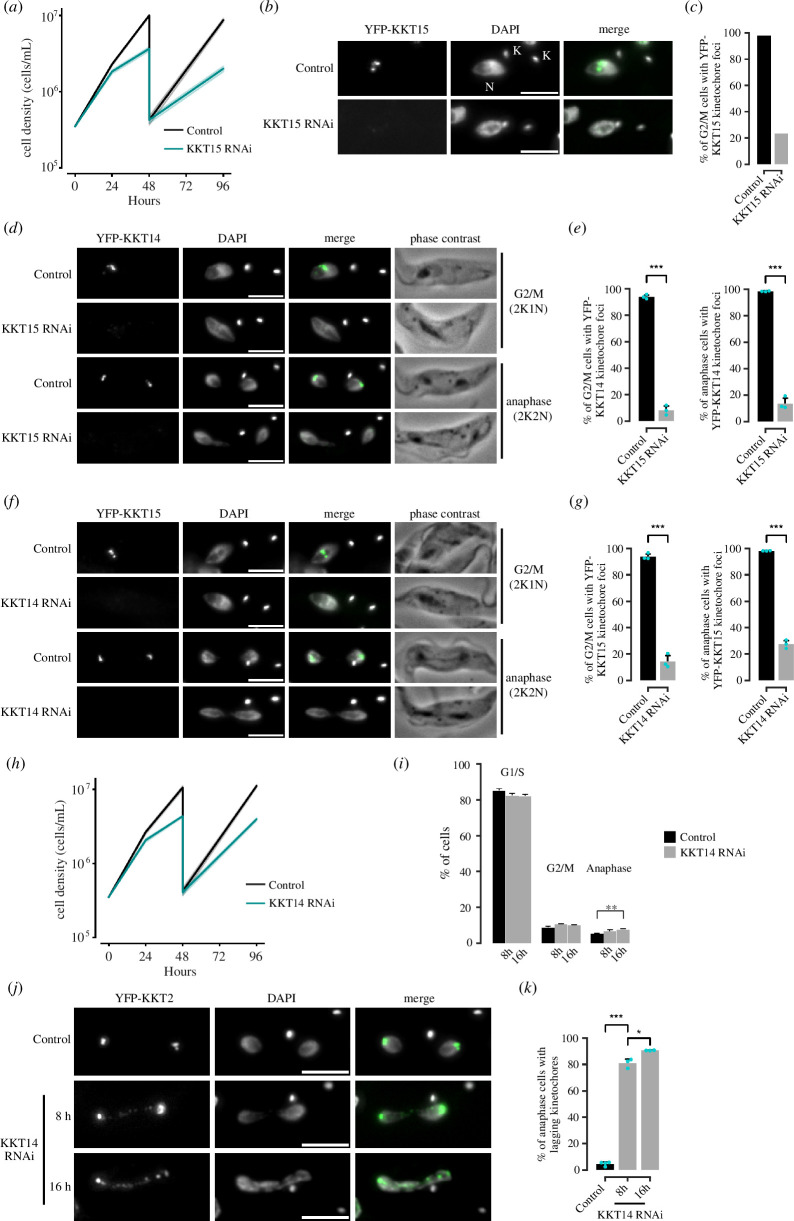
KKT14 is essential for accurate chromosome segregation in trypanosomes. (*a*) Growth curve upon RNAi-mediated knockdown of KKT15 using an RNAi construct against its 3’ UTR. Data are presented as the mean ± s.d. of three replicates. RNAi was induced with 1 μg ml^−1^ doxycycline and cultures were diluted on day 2. Cell line: BAP2533. (*b*,*c*) Validation of KKT15 knockdown. RNAi was induced with 1 μg ml^−1^ doxycycline and cultures were diluted on day 2. K and N stand for the kinetoplast and nucleus, respectively. Cell line: BAP2535. At least 130 cells per condition were quantified. (*d*) Representative fluorescence micrographs showing the localization of YFP-KKT14 upon RNAi-mediated knockdown of KKT15 in G2/M (2K1N) and anaphase (2K2N) cells. RNAi was induced with 1 μg ml^−1^ doxycycline for 24 h. Cell line: BAP2533. (*e*) Quantification of 2K1N and 2K2N that have kinetochore-like dots of YFP-KKT14 upon RNAi-mediated depletion of KKT15. All graphs depict the means (bar) ± s.d. of three replicates (shown as dots). (*f*) Representative fluorescence micrographs showing the localization of YFP-KKT15 upon RNAi-mediated knockdown of KKT14 in 2K1N and 2K2N cells. RNAi was induced with 1 μg ml^−1^ doxycycline for 24 h. Cell line: BAP2534. (*g*) Quantification of 2K1N and 2K2N that have kinetochore-like dots of YFP-KKT15 upon RNAi-mediated depletion of KKT14. All graphs depict the means (bar) ± s.d. of three replicates (shown as dots). A minimum of 50 cells per replicate were quantified in each condition. (*h*) Growth curve upon RNAi-mediated knockdown of KKT14. Data are presented as the mean ± s.d. of three replicates. RNAi was induced with 1 μg ml^−1^ doxycycline and cultures were diluted at day 2. Cell line: BAP2534. (*i*) Cell cycle profile upon knockdown of KKT14. RNAi was induced with 1 μg ml^−1^ doxycycline and cells were fixed at 8 or 16 h. All graphs depict the means (bar) ± s.d. of three replicates. A minimum of 700 cells per replicate were quantified. Cell line: BAP680. (*j*) Representative fluorescence micrographs showing lagging kinetochores (marked by tdTomato-KKT2) in anaphase cells upon KKT14 knockdown at 8 and 16 h post-induction. RNAi was induced with 1 μg ml^−1^ doxycycline. Cell line: BAP680. (*k*) Quantification of lagging kinetochores in anaphase cells upon KKT14 knockdown. All graphs depict the means (bar) ± s.d. of three replicates (shown as dots). A minimum of 50 cells per replicate were quantified in each condition. *p < 0.05, **p ≤ 0.01, ***p ≤ 0.001 (two-sided, unpaired *t*‐test).

We next performed an RNAi-mediated knockdown of KKT14 and KKT15 to assess their function for chromosome segregation. The RNAi construct for KKT14 is previously described [65], while that for KKT15 was established in this study (figure 7*a*–c). We found that kinetochore localization of KKT14 and KKT15 are mutually co-dependent (figure 7*d*–*g*), further supporting the notion that they form a complex. Although KKT14 depletion caused severe growth defects (figure 7*h*) [65], we failed to find obvious cell cycle profile changes at 8 or 16 h after induction of KKT14 RNAi, apart from a moderate increase in anaphase cells (figure 7*i*). In contrast, we observed lagging kinetochores in almost all anaphase cells even at 8 h post-induction (figure 7*j,k*). These results show that KKT14 is essential for accurate chromosome segregation and cell growth.

## 3. Discussion

Previous studies have shown that *T. brucei* has a Mad2-like protein, Cdc20 and components of the APC/C [66,67]. However, the Mad2-like protein localizes near basal bodies, not kinetochores, while Cdc20 lacks a well-conserved Mad2-interacting motif, suggesting that these proteins are unable to play a role in the canonical spindle checkpoint control [68,69]. Indeed, trypanosome cells cannot halt cell cycle progression in response to spindle defects [70]. By contrast, forced stabilization of cyclin B or treatment with proteasome inhibitors cause the nucleus to arrest in metaphase [70,71], raising a possibility that trypanosomes may possess an intrinsic mechanism that regulates the timing of nuclear division by controlling the APC/C^Cdc20^ activity in a Mad2-independent manner.

In this study, we propose that the kinetoplastid kinetochore proteins KKT14 and KKT15 are divergent Bub1/BubR1 and Bub3 orthologues, respectively. The discovery of a kinase fold in KKT14 was surprising because our previous sequence-based approach or a previous study that comprehensively catalogued pseudokinases failed to identify KKT14 as a pseudokinase [59]. In other words, KKT14’s pseudokinase domain is highly divergent from other kinases or pseudokinases. It is therefore remarkable that KKT14 retains significant structural similarities to the kinase domain of Bub1, including its N-terminal extension. In human Bub1, this N-terminal extension acts as a ‘minicyclin’ by making extensive contacts with the N-lobe of the kinase domain and thereby promoting an active conformation [48]. Similarly, in both the crystal structure of *A. spiralis* KKT14 and the AlphaFold2-predicted structure of *T. brucei* KKT14, the N-terminal extension makes extensive contacts with the N-lobe, which may stabilize the pseudokinase structure. Although the function of the KKT14 pseudokinase domain remains unclear, it is striking that all known KKT14 orthologs in kinetoplastids conserved it.

In contrast to KKT14, KKT15 has a readily discernible, common protein fold, namely a WD40 repeat beta-propeller. Our phylogenetic analysis places KKT15 close to Bub3. Taking into account also its interaction with the divergent Bub1/BubR1 protein KKT14, we propose that KKT15 is a Bub3 orthologue. Yet, its sequence is quite divergent from Bub3, particularly in trypanosomatids. In humans, Bub3 localizes at kinetochores by recognizing the phosphorylated KNL1 protein, which is not found in kinetoplastids. Furthermore, it remains unclear whether KKT15 binds a phosphorylated peptide because the regions of Bub3 that bind KNL1’s phosphorylated MELT motif [12] are not well conserved in KKT15 (electronic supplementary material, figures S4 and S5). It therefore remains unknown how the KKT14–KKT15 complex is recruited to kinetochores. Depletion of KKT2 disrupts KKT14 localization, suggesting that KKT14 and KKT15 are downstream of KKT2 [65]. Although our mass spectrometry data support a possibility that KKT14 and/or KKT15 directly interact with KKT2, AlphaFold2 fails to predict interactions between them. It will be important to identify direct interaction partners for KKT14 and KKT15 to reveal how these proteins function at kinetoplastid kinetochores.

In humans and *C. elegans*, some of the ABBA motifs in Bub1/BubR1 function by promoting kinetochore localization of Cdc20 and contribute to the strength of the checkpoint [19,72–75]. In trypanosomes, kinetochore localization of Cdc20 has not been reported [61] (and our unpublished data), and it remains unclear if, how, when, and where the ABBA motif of KKT14 may regulate the activity of Cdc20 and contribute to the cell cycle control. Addressing these questions will be key to understanding how KKT14 and KKT15 contribute to accurate chromosome segregation in trypanosomes that lack a canonical spindle checkpoint.

## 4. Material and methods

### 4.1. Trypanosomes and microscopy

All trypanosome cell lines used in this study were derived from *T. brucei* SmOxP927 procyclic form cells (TREU 927/4 expressing T7 RNA polymerase and the tetracycline repressor to allow inducible expression) [76] and are listed in electronic supplementary material, table S1. Cells were grown at 28°C in SDM-79 medium supplemented with 10% (v/v) heat-inactivated foetal calf serum, 7.5 µg ml^−1^ hemin [77], and appropriate drugs. Endogenous YFP tagging was performed using the pEnT5-Y vector [78] or a PCR-based method [79]. Endogenous 3FLAG-6HIS-YFP tagging was performed using pBA106 [70], while endogenous tdTomato tagging was performed using pBA892 [80]. LacO-LacI tethering experiments were performed as described previously using the LacO array inserted at the rDNA locus [80,81]. Inducible expression of GFP-NLS fusion and GFP-NLS-LacI fusion proteins was carried out using pBA310 [42] and pBA795 [80], respectively. Cell growth was monitored using a CASY cell counter (Roche). Expression of GFP fusion proteins and RNAi were induced with doxycycline at a final concentration of 10 ng ml^−1^ and 1 µg ml^−1^, respectively. All plasmids were linearized by *Not*I and transfected into trypanosomes by electroporation. Transfected cells were selected by the addition of 30 μg ml^−1^ G418 (Sigma), 50 μg ml^−1^ hygromycin (Sigma), 5 μg ml^−1^ phleomycin (Sigma) or 10 μg ml^−1^ blasticidin S (Insight Biotechnology).

### 4.2. Immunoprecipitation

For each experiment, 400 ml cultures of asynchronously growing cells (unless otherwise indicated) were grown to ~1 × 10^7^ cells ml^−1^ and harvested. Ectopic expression of GFP-tagged KKT14N^2–357^ and KKT14C^358–685^ in *T. brucei* was induced with 10 ng ml^−1^ doxycycline for 24 h. YFP-KKT14 and YFP-KKT22 were expressed from the endogenous locus. Where indicated, 10 µM MG132 (to arrest cells prior to anaphase) or 2 µM 1NM-PP1 (to inhibit the AUK1 kinase activity) were added for 4 h prior to harvesting the cells. Note that there was no noticeable change in the amount of co-purifying proteins in these conditions, so we pooled all results for the volcano plot analysis (figure 5*a* and electronic supplementary material, table S5). Immunoprecipitation of GFP/YFP-tagged proteins was performed with anti-GFP antibodies (11814460001, Roche) using a method we previously described [80]. 3FLAG-6HIS-YFP-tagged proteins expressed from the endogenous locus were immunoprecipitated using anti-FLAG M2 antibodies (F3165, Sigma) [82] and eluted with 0.5 mg ml^−1^ 3×FLAG peptide (F4799, Sigma) in BH0.15 (25 mM HEPES pH 8.0, 2 mM MgCl_2_, 0.1 mM EDTA pH 8.0, 0.5 mM EGTA pH 8.0, 1% NP-40, 150 mM KCl and 15% glycerol) supplemented with protease inhibitors (10 μg ml^−1^ leupeptin, 10 μg ml^−1^ pepstatin, 10 μg ml^−1^ E-64 and 0.2 mM PMSF) and phosphatase inhibitors (1 mM sodium pyrophosphate, 2 mM Na-β-glycerophosphate, 0.1 mM Na_3_VO_4_, 5 mM NaF and 100 nM microcystin-LR) with agitation for 25 min at room temperature. FLAG eluates were run on an SDS-PAGE gel, which was stained with Sypro-Ruby (Thermo Fisher).

### 4.3. *In vitro* kinase assay

To examine auto-phosphorylation activities in the immunoprecipitated 3FLAG-6HIS-YFP-KKT3/4/14/15 samples, 10 µl of FLAG eluates were mixed with 2.5 µl of 10× kinase buffer (500 mM Tris–HCl pH 7.4, 10 mM DTT, 250 mM β-glycerophosphate, 50 mM MgCl_2_, 50 μCi [^32^P] ATP and 100 μM ATP) in 25 µl volumes. The mixture was incubated at 30°C for 30 min, and the reaction was stopped by the addition of the LDS sample buffer (Thermo Fisher). The samples were run on an SDS-PAGE gel and stained with Coomassie Brilliant Blue R-250 (Bio-Rad) (not shown), which was subsequently dried and used for autoradiography using a phosphorimager screen. The signal was detected by an FLA 7000 scanner (GE Healthcare).

### 4.4. Mass spectrometry

Reduction of disulfide bridges in cysteine-containing proteins was performed with 10 mM DTT dissolved in 50 mM HEPES, pH 8.5 (56°C, 30 min). Reduced cysteines were alkylated with 20 mM 2-chloroacetamide dissolved in 50 mM HEPES, pH 8.5 (room temperature, in the dark, 30 min). Mass spectrometry samples were prepared using the SP3 protocol [83], and trypsin (Promega) was added in a 1:50 enzyme-to-protein ratio for overnight digestion at 37°C. Next day, peptide recovery was done by collecting supernatant on a magnet and combining with a second elution of beads with 50 mM HEPES, pH 8.5. For a further sample clean up, an OASIS HLB µElution Plate (Waters) was used. The samples were dissolved in 10 µl of reconstitution buffer (96:4 water:acetonitrile, 1% formic acid) and analysed by LC-MS/MS using QExactive (Thermo Fisher) in the proteomics core facility at EMBL Heidelberg (https://www.embl.org/groups/proteomics/). Peptides were identified by searching tandem mass spectrometry spectra against the *T. brucei* protein database with MaxQuant (v. 2.0.1) with carbamidomethyl cysteine set as a fixed modification and oxidization (Met), phosphorylation (Ser, Thr and/or Tyr) and acetylation (N-term and Lys) set as variable modifications. Up to two missed cleavages were allowed. The first peptide tolerance was set to 10 ppm (protein FDR 1%). Proteins identified with at least two peptides were considered significant and reported in electronic supplementary material, table S5. All raw mass spectrometry files and the custom database file used in this study have been deposited to the ProteomeXchange Consortium via the PRIDE partner repository [84,85] with the dataset identifier PXD047806.

Differential enrichment analysis of YFP-KKT14 versus YFP-KKT22 was performed on iBAQ values using the DEP package in R [86] (electronic supplementary material, table S5). Reverse hits and contaminants were removed, and results were filtered for proteins that were identified in all replicates of at least one condition. The data were background corrected and normalized by variance stabilizing transformation (vsn). Missing values were imputed using the k-nearest neighbour approach (knn). Potential interactors were determined using *t*-tests, with threshold values set to lfc = 2 and alpha = 0.05. The volcano plot shown was constructed using the EnhancedVolcano package [87].

### 4.5. Expression and purification of *Apiculatamorpha spiralis* KKT14C

To make pBA2356 (6HIS-KKT14^365–640^ from *Apiculatamorpha spiralis* (clone PhF-6)), the DNA was amplified from BAG142 (a synthetic DNA that encodes *A. spiralis* KKT14, codon optimized for expression in *E. coli*) with primers BA3187/BA3188 and cloned into RSFDuet-1 using *Bam*HI/*Eco*RI sites with the NEBuilder HiFi DNA Assembly kit (NEB) (electronic supplementary material, table S1). *Escherichia coli* BL21(DE3) cells were transformed with ~100 ng of plasmid DNA (pBA2356) and inoculated into 50 ml of 2×TY medium containing 50 μg ml^−1^ kanamycin and grown overnight at 37°C. The next morning, 6 l of 2×TY medium with 50 μg ml^−1^ of kanamycin was warmed at 37°C, and 5 ml of the overnight culture was inoculated into each litre. Cells were grown at 37°C with shaking (200 rpm) until the OD_600_ reached ~0.6. Protein expression was induced with 0.2 mM IPTG for 16 h at 20°C. Cells were spun down at 3400*g* at 4°C and resuspended in 200 ml of lysis buffer (50 mM sodium phosphate, pH 7.5, 500 mM NaCl and 10% glycerol) supplemented with protease inhibitors (20 μg ml^−1^ leupeptin, 20 μg ml^−1^ pepstatin, 20 μg ml^−1^ E-64 and 0.4 mM PMSF), benzonase nuclease (500 U per 1 l culture) and 0.5 mM TCEP. All subsequent steps were performed at 4°C. Bacterial cultures were mechanically disrupted using a French press (1 passage at 20 000 psi) and the soluble fraction was separated by centrifugation at 48 000*g* for 30 min. Supernatants were loaded on 5 ml of TALON beads (Takara Bio) pre-equilibrated with the lysis buffer. Next, the beads were washed with 300 ml of the lysis buffer with 0.5 mM TCEP, and proteins were eluted with 50 mM sodium phosphate pH 7.5, 500 mM NaCl, 10% glycerol, 250 mM imidazole and 0.5 mM TCEP. To cleave off the His-tag, samples were incubated with TEV protease in 1:50 (w/w) ratio overnight while being buffer-exchanged into 25 mM sodium phosphate, 250 mM NaCl, 5% glycerol, 5 mM imidazole and 0.5 mM TCEP by dialysis. To increase the sample purity and remove the His-tag, samples were re-loaded on TALON beads pre-equilibrated with the dialysis buffer and the flow-through was collected. Next, the sample was concentrated using 10-kD MW Amicon concentrator (Millipore), and loaded on Superdex 75 16/600 (GE Healthcare) columns to further purify and buffer exchange into 25 mM HEPES pH 7.5, 150 mM NaCl with 0.5 mM TCEP. Fractions containing the protein of interest were pooled, concentrated to 15.1 mg ml^−1^ using a 10-kD MW Amicon concentrator (Millipore), and flash-frozen in liquid nitrogen for −80°C storage.

### 4.6. Crystallization trials and structural determination

All crystals were obtained in sitting drop vapour diffusion experiments in 96-well plates, using drops of overall volume 200 nl, mixing protein and mother liquor in a 1:1 (v/v) ratio. Crystals of *A. spiralis* KKT14^365−640^ (15.1 mg ml^−1^) were grown at 4°C in MIDAS HT-96 B1 solution (Molecular Dimensions) containing 0.1 M sodium formate and 20% (w/v) SOKALAN CP 45. Crystals were briefly transferred into mother liquor prepared with the addition of 25% glycerol prior to flash-cooling by plunging into liquid nitrogen. Data collection and model-building X-ray diffraction data from *A. spiralis* KKT14^365−640^ were carried out at the I03 beamline at the Diamond Light Source (Harwell, UK). The structure was solved using the AlphaFold2-predicted structure of *A. spiralis* KKT14^398−640^ as a model with a molecular replacement software, PHASER [88], followed by initial model building with BUCCANEER [89]. The data were scaled to 2.2 Å based on I/Iσ parameters (I/Iσ value of 2.0 was used as a threshold). Further manual model building and refinement were completed iteratively using COOT [90] and PHENIX [91]. All images were made with PyMOL (v. 2.5.2, Schrödinger). Protein coordinates have been deposited in the RCSB protein data bank with the accession number 8QOH.

### 4.7. Bioinformatic analysis of KKT14 and KKT15

The protein sequences for KKT14 and KKT15 were retrieved from the TriTryp database [92] or published studies [93,94]. Searches for their homologous proteins were done using BLAST in the TriTryp database [92] or manual searches using hmmsearch (HMMER v. 3.0) on predicted proteomes using manually prepared hmm profiles [95]. Multiple sequence alignments were performed with MAFFT (L-INS-i method, v. 7) [96] and visualized with the clustalx colouring scheme in Jalview (v. 2.11) [97]. The pairwise sequence identity and similarity between *A. spiralis* KKT14^365−640^ and *T. brucei* KKT14^358–685^ were calculated using EMBOSS Needle [98]. Structures and interactions were predicted with AlphaFold2-Multimer-v. 2.3.1 [99,100] through ColabFold v. 1.5.3 using MMseqs2 with 24 recycles (UniRef + Environmental) [101]. The rank 1 model, predicted aligned error and pLDDT plots for each prediction are provided in the electronic supplemental material (dataset S1–S4). In all cases, similar results were obtained for five predictions. All structure figures were made using PyMOL v. 2.5.2 (Schrödinger, LLC). The following command was used to map the pLDDT score onto the AlphaFold2-predicted structure models: spectrum b, rainbow_rev, maximum = 100, minimum = 50. Foldseek searches were carried out using the Web server against AlphaFold2-predicted structure databases covering UniProt50, Swiss-Prot and Proteome (v. 4, Mode 3Di/AA) (https://search.foldseek.com/search) [52,102].

### 4.8. Phylogenetic analysis of KKT15 and related WD40 repeat-containing proteins

To conduct a phylogenetic analysis to determine to which WD40 repeat proteins KKT15 proteins are closely related, we selected the 30 best EggNOG KOG/COG hits resulting from online HHpred [60,103] of our best, most sensitive KKT15 multiple sequence alignment. The latter was obtained by iterative profile HMM searches combined with phylogenetic analysis among a local database of euglenozoa and some other eukaryotic protein sequences, as well as a local eukaryote-wide dataset [104] (electronic supplementary material, table S3). The trusted list of KKT15 orthologues across these euglenozoa was aligned using MAFFT (v. 7.505, option L-INS-i, alignment and profile HMM are included in electronic supplementary material, dataset S5) [96] and submitted to online HHpred to search in its COG_KOG_v. 1.0 database. Among these 30 best hits, there were only KOG families, and one duplicate, which we removed (electronic supplementary material, table S4). We collected the corresponding profile HMMs of these KOGs from EggNOG (v. 5.0) [105] and added our initial KKT15 HMM. We then executed hmmscan (hmmer.org) of these HMMs versus a subsampled (49 diverse eukaryotes) version of our local eukaryotic database, applying an *E*-value cut-off of 1 × 10^–5^. We assigned the retrieved eukaryotic proteins to their respective KOG (or KKT15), and appended the original euglenozoan KKT15 orthologues to that family as well. We subsequently, again for all KOGs/KKT15, used hmmsearch among the assigned hits, in order to be able to retrieve the 100 best hits per KOG/KKT15, and gathered of these 100 hits only the domain that was hit by the HMM (the putative WD40 repeat). For Rae1 (KOG0647), we also included the hit regions of some additional Discoba orthologue candidates, because these were not part of the 49 species selection but potentially informative in placing KKT15. Except for KKT15 and these additional Discoba sequences, we subjected the KOG sequence selections to CD-hit (v. 4.8.1, identity cut-off 70%) [106], in order to facilitate the phylogenetic analysis and interpretation. We then removed all sequences across all KOGs that were shorter than 50 amino acids (i.e. this was not applied to KKT15). Each KOG was separately aligned using MAFFT (L-INS-i).

We then employed two different strategies to combine all sequences into a single alignment, resulting in two different phylogenies (referred to as tree 1 and tree 2, found in electronic supplementary material, figure S3*a*,*b*, respectively). For tree 1, we similarly aligned KKT15, and subsequently used MAFFT’s –merge option to combine all individual alignments into a single alignment (parameters: --localpair --maxiterate 100 --merge). We also had identified five additional, potentially close KKT15 homologues of different species, which cannot be evidently classified as a particular WD40 repeat protein. We added them to the alignment using MAFFT option –add (parameters: --maxiterate 1000 --add). For tree 2, we used MAFFT –merge (parameters: --localpair --maxiterate 100 –merge) on just the KOG’s individual alignments, and subsequently used MAFFT –add (parameters: --maxiterate 1000 –add) to add KKT15 orthologues and the sequences of unknown identity to it. The alignments for tree 1 and tree 2 hence differ in the way the KKT15 sequences are aligned: either first among one another (tree 1), or only through adding the sequences to the already existing (merged) alignment of the KOGs. The first approach forces the KKT15 sequences to be monophyletic in the tree, while the second approach does not. We trimmed both alignments using trimAl [107] (v. 1.4.rev15, option -gappyout) and removed sequences with >85% gaps. We used the alignments to infer a maximum likelihood phylogeny with IQ-TREE (v. 2.0.3) [108], applying an evolutionary model selected by ModelFinder [109], also allowing for complex mixture (C-series) models to be selected. Branch support was estimated through ultrafast bootstraps (1000 replicates) [110]. The tree with the highest likelihood, either the maximum likelihood tree or the consensus tree, was selected for visualization in iTOL [111]. The phylogenies were initially rooted in a well-supported clade with a relatively long branch, which was not closely associated with Rae1, Bub3 or KKT15 (KOG2111). The phylogenies were annotated using, if available, the name of the human protein belonging to each KOG. If not available, the budding yeast protein name was used. The multiple sequence alignments and raw IQ-TREE output can be found in electronic supplementary material, dataset S5. The full, uncollapsed phylogenies can be inspected on iTOL: https://itol.embl.de/tree/62145194227274281702966749 (tree 1, associated with electronic supplementary material, figure S3*a*) and https://itol.embl.de/tree/62145194227421141702968325 (tree 2, associated with electronic supplementary material, figure S3*b*). Note that alongside KKT15, we performed a similar online HHpred search for a refined KKT14 multiple sequence alignment, the results of which are also reported in electronic supplementary material, table S4. The alignments used as input for these searches can be found in electronic supplementary material, dataset S6.

## Data Availability

All raw mass spectrometry files and the custom database file used in this study have been deposited to the ProteomeXchange Consortium via the PRIDE partner repository [84,85] with the dataset identifier PXD047806. Protein coordinates have been deposited in the RCSB protein data bank with the accession number 8QOH. Supplementary material is available online [112].
